# News and Events of the Week

**Published:** 1910-05-14

**Authors:** 


### News and Events of the Week.

Mr. John Nettleton Terry, M.R.C.S., of Mirfield, Yorkshire, who died on March 20, at the age of 77, left estate valued at £57,567 8s. 10d. gross and £30,947 10s. 7d. net.

A debate on vaccination has been arranged between Dr. J. Lionel Tayler (University Extension Lecturer) and Mr. John H. Bonner, to take place at the Caxton Hall, Westminster, on Tuesday, May 24, 7.30 p.m.

The late Surgeon-General Charles Dodgson Madden, C.B., of Woodside, The Green, St. Leonard's-on-Sea, Sussex, who served during the Crimean War and also in India during the Mutiny, and who died on January 5 last at the age of 76, has left estate valued at £4,043 gross.

Until comparatively recent years a sea holiday was more or less a monotonous voyage to a definite destination, and probably involved a change of vessel for the return journey; but the pleasure cruises which are organised by the shipping companies now combine health-giving with sight-seeing all over the Old World. The Orient Steam Navigation Company will have three of its vessels engaged in pleasure-cruising this season. Its new giant steamer *Otranto*, 12,124 tons, will make an attractive Whitsuntide cruise to Portugal, Spain, and the Mediterranean, occupying seventeen days; while the *Ophir* and *Omrah* are to make a series of thirteen-day cruises to Norway and the Baltic capitals in June, July, and August. A series of handbooks dealing with these cruises can be obtained from the company.

Messrs. J. and A. Churchill have a new edition of Vol. 2 of *Allen's Commercial Organic Analysis* just ready for publication. This volume has been re-written under the editorship of Dr. H. Leffmann and Mr. W. A. Davis, B.Sc. The subjects and authors are as follows: "Fixed Oils, Fats, and Waxes," by C. A. Mitchell; "Special Characters and Methods," by L. Archbutt; "Butter Fat," by C. Revis and E. R. Bolton; "Lard," by C. A. Mitchell; "Linseed Oil," by C. A. Klein; "Higher Fatty Acids," by W. Robertson; "Soap," by H. Leffmann; "Glycerol," by W. A. Davis; "Cholesterols," by J. A. Gardner; "Wool Fat and Cloth Oils," by A. H. Gill. The same firm also has in hand a *Post-mortem Manual*: an illustrated handbook of morbid anatomy and post-mortem treatment, by C. R. Box, M.D., F.R.C.S., F.R.C.P., physician to out-patients, St. Thomas's Hospital.

The Medical Mission Auxiliary of the Church Missionary Society held its annual meeting in the Queen's Hall on Friday last week, Professor A. Carless, M.S., F.R.C.S., of King's College Hospital, in the chair. The annual report was read by the Rev. R. Elliott, M.A., L.R.C.S.I., Secretary to the Medical Committee, and showed that the Society now maintains fifty hospitals in various parts of the world, with a total of 3,075 beds, in which over 29,000 patients were treated last year, besides which over 1,100,000 visits of out-patients were recorded. The Chairman drew attention to the magnitude of this work, to the economical administration (the expenditure being less than £40,000) and to the high qualifications of the men engaged in it—one of the missionaries present on the platform (Dr. Pennell) was a gold medallist—and all were men who would have been in the front rank of the profession at home. The meeting was also addressed by Messrs. B. van Someren Taylor, M.B., C.M. (Edin.), of Hing-hwa, China; T. L. Pennell, M.D. (Lond.), F.R.C.S., of Bannu, on the north-west frontier of India; and J. H. Cook, M.S. (Lond.), F.R.C.S., D.T.M., of Mengo (Uganda), who gave some account of their work as medical missionaries.

The Local Government Committee of the London County Council suggests that a tablet be affixed to 88 Paradise Street, Rotherhithe, to commemorate the residence thereat of Thomas Henry Huxley. The Council has already decided to commemorate Huxley's residence at 4 Marlborough Place, St. John's Wood, but it is pointed out that very few opportunities occur of indicating houses of historical interest in such districts as Rotherhithe.

Before Parliament broke up Sir William Collins asked the Secretary of State for the Home Department whether it was proposed to introduce legislation during the present Session to give effect to the recommendation of the Departmental Committee appointed to inquire into the law relating to coroners and coroners' inquests, and into the practice in coroners' courts. Mr. Churchill, in reply, stated that he had under consideration two valuable reports made by this Committee, but feared that as matters now stand no pledge could be given as to when it will be possible to propose legislation to carry out its recommendations.

The Australian Women's National League (political) has formed an ancillary body, named the Red Cross League, for women's share in defence. The women are to study first aid (under the St. John Ambulance Association), camp cookery, and signalling.

The New Gallery Restaurant and Wiener Café, which occupies in Regent Street the site of the New Gallery, will be opened to the public on May 23. Its cuisine will be Austrian. Gottlieb's Orchestra has been engaged by the proprietors to play daily.

The annual general meeting of the Guild of the Royal London Ophthalmic Hospital was held last week at 66 Portland Place, W., by kind permission of Mrs. Sidney Bristowe. The chair was taken by the Hon. Mrs. Richard Grosvenor, and the report for 1909 read by Mrs. W. F. Lister (hon. secretary). It described the many activities of the Guild in supplying comforts for the patients in Moorfields Eye Hospital. Mrs. W. A. Whitworth, in moving the adoption of the report, said that it was the privilege of this Guild to help in saving others from becoming blind. The necessary treatment of affections of the eye was provided at the hospital, and it was very often through the lack of common necessities that a ward was closed. Surgeons were afraid to send home children treated at the hospital because of the danger of septic poisoning in the homes of the poor; with more money much good work might be done. Mr. Stephen Paget said that the Guild had nearly 300 members, seven local centres, and had contributed over £2,000 since 1900 towards the work of the hospital. Mr. Arnold Lawson also spoke. Mr. H. P. Sturgis, chairman of the Committee of Management of the hospital, said that twenty beds were now vacant for want of funds.

A Council meeting of the Association of Medical Officers of Health was held at the Holborn Restaurant on April 28, and, in the unavoidable absence of Dr. Crookshank, Dr. Rashell Davison was voted to the chair. The Treasurer (Dr. Gibb-Smith) reported that many new members had joined the Association since the last meeting of the Council, and there was a good balance in hand after all the expenses had been paid. The Secretary (Dr. Belilios) stated that Dr. Freemantle, county medical officer for Hertfordshire, would read a paper in the Public Health Section of the British Medical Association on "The Influence of Part-time Medical Officers of Health on Public Health," and the Council expressed the hope that the section would be well attended. It was noted that the Rochdale, Richmond, and Salford divisions of the British Medical Association would instruct their delegates to vote for the rescission of minute 234 at the representatives' meeting, and that the Salford division would move: "That in every form of public medical service the system of part-time appointments of medical practitioners should be maintained as far as such appointments can be made consistently with the requirements of the services, and that all public medical officers should be protected in carrying out their duties, should be secured from capricious dismissal or reduction of salary, and should be allowed to participate in any superannuation scheme approved by a Local Government Board." It was decided to recommend those members who belong to both Associations to move so that this resolution would be supported by their delegates at the representative meeting of the British Medical Association. Dr. Davison informed the meeting that a paper was to be read at the meeting of the Urban District Councils Association next July on the advantages of the present part-time public health service.

By the kindness of the Duchess of Marlborough the annual meeting of the Invalid Children's Aid Association (London) Incorporated, 69 Denison House, 296 Vauxhall Bridge Road, S.W., was held on April 27, in Sunderland House. The chair was taken by her Grace, who pleaded the needs of the Association with much force. She reported a new departure in the work of the Association by the acquisition of a special home, for which the sum of £500 a year would be required. Towards this amount she promised £200 if a further £300 could be raised. The Bishop of Bath and Wells followed. He dwelt on the need of personal service. Mr. Robert Hutchinson and Mr. Darcy Power spoke on the medical aspect of the Association, and Mrs. Carl Meyer, as a member of a Care Committee, made an appeal for workers and funds for the East End. Miss MacNaughtan enlarged on the need of more visitors for the invalid children. The meeting, which was largely attended, adopted the report of the Society, and votes of thanks were passed to her Grace and the speakers.

At the annual meetings of the Women's Total Abstinence Union, the Nurses' Total Abstinence League and the Certified Midwives' Total Abstinence League gave interesting reports of work. The Nurses' League reports a membership of 443. It is encouraging to note that the attitude of the nursing profession towards the question of total abstinence becomes increasingly sympathetic, and this is without doubt due, in part, to the fact that scientific and medical evidence speak so clearly in its favour. The League has held several meetings in hospitals, and drawing-room meetings have been given by the Dowager Lady de Rothschild, Mrs. Pearce Gould, and other friends. A branch of the League exists in Birmingham with seventy-eight members. The Certified Midwives' League was founded by Dr. Mary Rocke in 1905 and affiliated to the Women's Total Abstinence Union in 1909. It has a membership of 460. Meetings have been held in drawing-rooms and at the Chapter House, St. Paul's Churchyard. Branches have been formed at Oxford and Nottingham. Particulars of both Leagues can be obtained from the Secretary, 4 Ludgate Hill, London, E.C.

Wherever the beautiful and accomplished Mary Queen of Scots was imprisoned, previous to laying her head with calm courage on the block at Fotheringay Castle, she managed to correspond with her friends, both at home and abroad. One of her letters, sent during her detention at Chatsworth, to her brother-in-law, Charles IX., in which she begged him to interecede with Elizabeth, and also to send troops to Scotland, sold for £715 on May 4 at Sotheby's. On May 6, two days later, Messrs. Puttick and Simpson sold a "medical recipe" written by David Livingstone, signed "D. L.," together with autograph letters of H. M. Stanley, Oliver Wendell Holmes, and John Ruskin, for 11s. the lot. The following is a copy of the recipe or prescription, as it should more properly be called: "Rx Potassæ Hydriod ʒss, Decoct Sarzæ ℥vj, M Ft Mist cap coch mag jj ter die. 6 Feb. 1840. Pro Guli Mason. D. L." This was probably written before Dr. Livingstone received his appointment from the London Missionary Society, and shortly after he passed the qualifying examination with honours, after many years of hard toil, overcoming difficulties, alone, without assistance, which most young men would have considered quite insurmountable. The records of his after-life illumine the page of history, and his body, "brought by faithful hands over land and sea," rests in Westminster Abbey, not far removed from the remains of the charming though unfortunate Queen.

